# The Potential of Honey as a Prebiotic Food to Re-engineer the Gut Microbiome Toward a Healthy State

**DOI:** 10.3389/fnut.2022.957932

**Published:** 2022-07-28

**Authors:** Kathleen R. Schell, Kenya E. Fernandes, Erin Shanahan, Isabella Wilson, Shona E. Blair, Dee A. Carter, Nural N. Cokcetin

**Affiliations:** ^1^Australian Institute for Microbiology and Infection, University of Technology Sydney, Sydney, NSW, Australia; ^2^School of Life and Environmental Sciences, University of Sydney, Sydney, NSW, Australia; ^3^Faculty of Medicine, Imperial College London, London, United Kingdom

**Keywords:** honey, medicinal honey, prebiotic honey, prebiotics, gut microbiome, gut health, dietary remediation

## Abstract

Honey has a long history of use for the treatment of digestive ailments. Certain honey types have well-established bioactive properties including antibacterial and anti-inflammatory activities. In addition, honey contains non-digestible carbohydrates in the form of oligosaccharides, and there is increasing evidence from *in vitro*, animal, and pilot human studies that some kinds of honey have prebiotic activity. Prebiotics are foods or compounds, such as non-digestible carbohydrates, that are used to promote specific, favorable changes in the composition and function of the gut microbiota. The gut microbiota plays a critical role in human health and well-being, with disturbances to the balance of these organisms linked to gut inflammation and the development and progression of numerous conditions, such as colon cancer, irritable bowel syndrome, obesity, and mental health issues. Consequently, there is increasing interest in manipulating the gut microbiota to a more favorable balance as a way of improving health by dietary means. Current research suggests that certain kinds of honey can reduce the presence of infection-causing bacteria in the gut including *Salmonella*, *Escherichia coli*, and *Clostridiodes difficile*, while simultaneously stimulating the growth of potentially beneficial species, such as *Lactobacillus* and *Bifidobacteria.* In this paper, we review the current and growing evidence that shows the prebiotic potential of honey to promote healthy gut function, regulate the microbial communities in the gut, and reduce infection and inflammation. We outline gaps in knowledge and explore the potential of honey as a viable option to promote or re-engineer a healthy gut microbiome.

## Introduction

Gut microbiota plays a critical role in human health and well-being by aiding digestion, synthesizing vitamins, stimulating the immune system, and protecting against enteropathogenic infections (1–3). Disruptions to the symbiotic relationships within the gut microbiota and with its host, known as dysbiosis, can result in the development and progression of numerous diseases, ranging from inflammatory bowel disease and colon cancer to allergies, obesity, and mental health issues (4–8). As the composition and function of the gut microbiome are significantly influenced by diet (9–13), there is considerable interest in manipulating it to a more beneficial balance through dietary means (1, 14, 15). Prebiotics, which are typically non-digestible carbohydrates and other foodstuffs, have been used to promote specific, favorable changes in the gut that confer health benefits to the host (16). These benefits have been associated with increased numbers of potentially beneficial microbes including bifidobacteria and lactobacilli in the gut, and/or increased production of metabolites like short-chain fatty acids (SCFA) by gut microbes (14).

Honey has a long history of use as a therapeutic agent, including as a tonic to promote good digestive health (17, 18). It is now scientifically established that honey has many therapeutic properties, including antibacterial, anti-inflammatory, wound healing, and antioxidant activities (19, 20). Certain kinds of honey are especially “bioactive,” and this has been linked predominantly to their floral source (21, 22). Honey contains non-digestible oligosaccharides, and growing evidence from *in vitro*, animal, and pilot human studies suggests that some kinds of honey could have prebiotic capability to induce beneficial changes in the gut. In this paper, we summarize the history and composition of honey as a therapeutic for digestive health, the effect of the gut microbiome on human health and how it can be shaped by diet and prebiotics, and finally, explore the current evidence for, and future potential of, the honey as a prebiotic.

## Honey as a Therapeutic Agent Throughout History

### Honey in the Human Diet and Its Use for Digestive Health Throughout History

The importance of honey in the diets of human foragers throughout history has been well documented. Honey, as well as residual bee larvae in wild honey, may have been an important source of energy, fat, and protein for early humans (reviewed in (23)). It has been suggested that routine consumption of honey, an energy-dense and easily digestible food source, to supplement meat and plant foods, may have played an important role in shifting the diet from a low-calorie to an energy-rich, calorie-dense diet to support increasing brain activity during the evolution of larger hominin brains (23–25). The reduction of molar size, indicating the consumption of foods requiring less mechanical breakdown, along with the documented use of Oldowan tools (50,000–10,000 BCE) that may have been used for honey collecting as denoted in rock art also support this idea (23).

Honey has a long history as a treatment for gastrointestinal conditions. Circa 25 AD, Roman physicians prescribed different types of honey as a cure for both diarrhea and constipation, and Islamic holy scripts dating back to the 8th century show the prophet Muhammad recommending the use of honey for diarrhea (26, 27). In various books and records from eastern Europe and Arab countries, the use of honey in the prevention and treatment of peptic ulcers, gastritis, and gastroenteritis is often reported (28).

Many modern studies into the digestive health benefits of honey have shown that ingesting honey shortens the duration of bacterial diarrhea in children (29) and in critically ill tube-fed patients who were also reported to be less likely to suffer from organ failure on honey treatment (30). Honey also improved the recovery of patients with viral gastroenteritis (31). Other studies suggest that honey has a protective effect on the stomach (32). The consumption of relatively large amounts of honey (50–100 g) can also have a mild laxative effect, due to insufficient absorption of the fructose in honey (27).

### The Composition and Therapeutic Properties of Honey

Honey is a naturally sweet substance produced by honey bees (*Apis mellifera*) from the nectar of flowers or from plant secretions. The composition of honey is complex with over 200 components, many of which are dependent on the floral source (28). The nectar collected by bees to make honey affects the flavor, color, and medicinal properties of different honeys (21). Honey is composed mostly of sugar (up to 80%) with the monosaccharides fructose and glucose making up the majority (∼70%), and di-, tri-, oligo-, and polysaccharides composing the remainder. Other components of honey include a water content of between 15 and 20%, proteins, organic acids (such as gluconic acid), minerals, plant phytochemicals, and vitamins (25, 33).

Honey has numerous nutritional and therapeutic benefits including antimicrobial, antioxidant, anti-inflammatory, and wound healing activities. Of these, the most extensively studied through *in vitro* and *in vivo* experiments and human trials has been antimicrobial activity (19, 22, 27, 34–37). The continued medicinal use of honey as a therapeutic agent can be attributed to its broad-spectrum antimicrobial properties, which have proven effective against many pathogenic organisms, including multi-drug resistant strains. The antimicrobial activity of honey is multi-factorial and is derived from osmolarity, acidity, the production of hydrogen peroxide, and the presence of non-peroxide factors (36). There have been no documented cases of microbial resistance to the inhibitory effects of honey and honey resistance cannot be induced (38–40). This is likely because honey has multiple mechanisms of antimicrobial action (41).

Relevant to the gut, honey inhibits undesirable microbes such as *Listeria monocytogenes* in milk, as well as *Clostridium perfringens* and *Eubacterium aerofaciens* (42). Additionally, honey also inhibits many enteropathogenic organisms, such as *Salmonella* species (multi-drug resistant strains); *Shigella* species; enteropathogenic *E. coli* (including multi-drug resistant strains), *Enterobacter* species, *Yersinia enterocolitica, Campylobacter* species, and *Clostridium difficile* (37, 43–49). Apart from its direct antibacterial activity, honey has been shown to prevent the attachment of *Salmonella* species to mucosal epithelial cells *in vitro*, thereby preventing the establishment of infection (50).

The antioxidant effect of honey is largely attributed to its phenolic compounds which, when ingested by an individual, can provide protection in the bloodstream and within cells (51). As with antimicrobial activity, the antioxidant capacity of honey is highly variable and dependent on floral sources. Generally, darker-colored honeys show higher levels of antioxidant activity than their lighter counterparts, as color is also determined by phenolic content. The phenolic content of honey has also been linked to its anti-inflammatory effects, and honey has been reported to downregulate pro-inflammatory cytokines, upregulate anti-inflammatory cytokines (52), and interrupt inflammation mediators (53, 54). Thus, the anti-inflammatory and antioxidant effects of honey are closely linked. The anti-inflammatory, antioxidant, antimicrobial, and wound healing properties of some honeys have been used extensively in the treatment of wounds, burns, and ulcers (20, 55–57); however less is known about their systemic effects when ingested.

## Diet and the Gut Microbiome

### The Gut Microbiome and Its Contribution to Human Health

The gut microbiome is recognized as playing a significant role in human health. Its composition varies significantly between individuals and within the same individual over time, influenced by factors such as age, sex, ethnicity, geographic location, medication usage, stress, gastrointestinal infections, smoking status, and diet (13, 58–61). Studies have implicated the gut microbiome in brain health and cognitive function, nervous system development and maturation, and the immune system and response, as well as asthma and allergies, cardiovascular health, and obesity (13, 14, 58, 59, 61–66). Consequently, there have been concentrated research efforts to identify a core ‘healthy’ human microbiome (58, 59, 67, 68).

Much of the earlier research was focused on profiling the microbiota of the gut to identify bacterial species and groups associated with beneficial outcomes—that is, probiotic species. Certain types of probiotic gut bacteria, such as bifidobacteria and lactobacilli, have been noted to lessen the severity of symptoms of rotavirus- and antibiotic-associated diarrhea in infants (69), aid in the breakdown of lactose in individuals with lactose intolerance, help with bile deconjugation, promote beneficial organic acid production, and compete with gastroenteritis-causing bacteria to prevent infection (70, 71). In contrast, an ‘unhealthy’ gut microbiome is linked to a reduction of beneficial bacteria, overgrowth of certain fungal species, increase in putrefactive bacteria, and increase in opportunistic pathogens (58). Although the association of specific commensal microbial types in health and disease is recognized, it is not always clear whether the microbes are the cause or effect (72, 73).

However, it is now more commonly accepted that a ‘healthy’ gut microbiome is one that performs desired metabolic functions and has a symbiotic relationship with its host, rather than only specific bacterial populations in greater or lesser numbers (58, 59, 74, 75). Molecular studies confirm that many genes encode for similar microbial functions across different bacterial species, including those associated with degradation and digestion of complex sugars, production of SCFA, energy production, and the synthesis of vitamins (59, 74, 76). A predominance of beneficial microbes, microbial activities, and resultant metabolites, acts to maintain a healthy gut barrier, facilitate immune homeostasis, and host metabolic health. Reductions in beneficial microbial activity in the gut, along with increased intestinal permeability, can increase interactions between microbial antigen and the immune system, triggering inflammatory processes both in the gut and systemically, and contribute to, or drive, poor host health (77). However, the ability to manipulate the gut microbiome using targeted nutritional approaches, which can reduce the severity of disease or improve health outcomes, is a key goal in translating an understanding of the gut microbiome into a therapeutic benefit (5, 73, 78).

### The Impact of Diet and Prebiotics on Gut Microbiota

Diet plays a significant role in the functioning and composition of the gut microbiome (14, 79). The impact of diet on the gut microbiome has been shown as early as infancy, where the composition and diversity of the microbiota of breast-fed and formula-fed infants differed significantly (80). Studies have shown that the gut microbiome may co-evolve with diet. A study comparing the diet and gut microbiota of children from Europe and a rural African village showed that the African microbiome had a depletion of Firmicutes and was enriched with Acinetobacteria, Bacteroidetes, and a specific abundance of *Xylanibacter* and *Prevotella* that could improve the ability to extract calories from the indigestible plant polysaccharides that contributed to the diet of the African children (10). Long-term dietary patterns, particularly protein and animal fat as compared to carbohydrate/fiber intake, are linked to the assemblage of the gut microbial community and associated with population-wide patterns such as the relative abundance of *Bacteroides* and *Prevotella* (81). While the adult microbial community is relatively stable over time and linked to long-term diet (82, 83), it is possible to alter both the compositional makeup and function of the gut microbiota through short-term dietary alteration (84, 85).

Prebiotic foods, such as non-digestible carbohydrates, do not get absorbed in the upper gut and reach the colon intact where they are readily available for use as a selective substrate by gut microbiota. This results in selective stimulation of beneficial microbial populations and functions in the gut (16, 86). Dietary prebiotics have been linked to health-promoting effects including immunostimulation, improved digestion and absorption, vitamin synthesis, reduced cholesterol, reduced gas distension, regulation of opportunistic and invading pathogen growth, improved mineral (especially calcium) absorption, modulation of lipid metabolism *via* fermentation products, anti-inflammatory activity, and decreased risk of cancer and cardiovascular disease (11, 14, 87–95). The importance of bacterial functions related to carbohydrate metabolism in the colon is well established (4, 96). Indigestible complex carbohydrates, oligosaccharides, polysaccharides, and peptides are major drivers of gut microbial composition and activity (97). As such, there is a great interest in identifying sources of these carbohydrates for use as prebiotics.

## The Prebiotic Potential of Honey

### Evidence From Laboratory Studies

**FIGURE 1:**
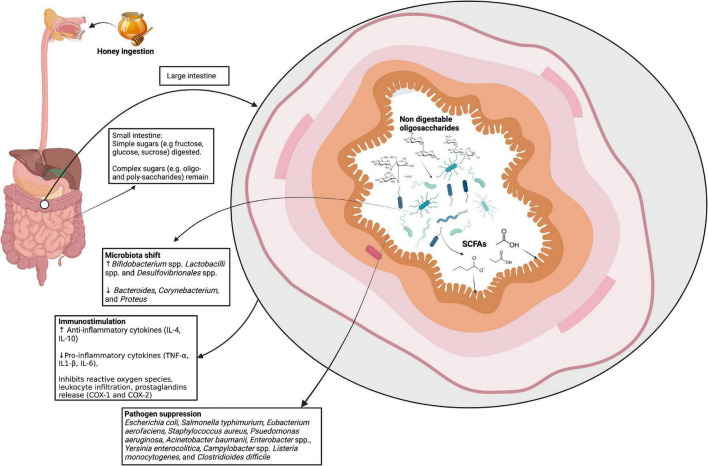
The proposed prebiotic effects of honey. Following ingestion, the simple sugars in honey are absorbed in the small intestine. The non-digestible components, including oligosaccharides, reach the lower intestines where they are proposed to be involved in immunostimulation, modulating the microbiota, and suppressing pathogens. SCFAs, short-chain fatty acids; IL, interleukin; TNF, tumor necrosis factor; COX, cyclooxegenase. Image created with BioRender.com.

Although honey is predominantly made up of simple sugars (monosaccharides) that are rapidly absorbed in the small intestine, there are also di-, tri-, and oligosaccharides that are present in smaller quantities (98, 99). These oligosaccharides and low-weight polysaccharides in honey are likely to resist degradation by host enzymes and are capable of reaching the lower gut to exert prebiotic effects (100). Many studies suggest a prebiotic effect of various kinds of honeys of different floral varieties (Table 1). The proposed prebiotic effects of honey, and honey oligosaccharides, are summarized in Figure 1.

**TABLE 1:** Summary of the studies showing prebiotic effects of various honeys.

Honey type and source	Experimental approach	Prebiotic effect reported	References
***in vitro* studies**			
Honeydew (Spain)	Fecal bacteria fermentation	Increase in beneficial lactobacilli and bifidobacteria, reduction in enteric bacteria and *Bacteroides*.	(83)
Buckwheat (China)	16S rDNA sequencing of V4 region	Increase in *Bifidobacterium* spp.	(84)
Juazeiro and Jurema-branca (Brazil)	Broth turbidity assay, with growth measured as turbidity	Increase in viable counts of *Bifidobacterium lactis* and *Lactobacillus acidophilus*	(85)
Manuka (New Zealand)	Microplate growth bioassay, with growth measured as optical density (turbidity)	Increase in *Lactobacillus reuteri, L. rhamnosus* and *Bifidobacterium lactis.* Inhibition of pathogenic bacteria: *Escherichia coli, Salmonella typhimurium*, and *Staphylococcus aureus*	(86)
Clover (United States)	Microbroth dilution, with growth measured as optical density (turbidity)	Increase in *Bifidobacterium longum*, *B. adolescentis*, *B. breve*, *B. bifidum*, and *B. infantis* Equally effective as commercial prebiotics: fructooligosaccharide, galactooligosaccharide, and inulin	(88)
Clover (United States)	Microbroth dilution, with growth measured as optical density (turbidity)	Increase in two commercial *Bifidobacterium* spp. strains (in skim milk supplemented with honey)	(93)
Sage, alfalfa and sourwood (United States)	Cultural enumeration (colony counts on agar plates)	Increase in *Streptococcus*, *Lactobacillus*, and *Bifidobacterium* strains	(92)
Acacia and chestnut (Saudi Arabia)	Agar disk diffusion assay, cultural enumeration (colony counts on agar plates)	Increase of *bifidobacteria* and *lactobacilli*, specifically by reducing doubling time Inhibition of pathogenic *Listeria monocytogenes*	(91)
Acacia and chestnut (Croatia)	Agar disk diffusion assay, cultural enumeration (colony counts on agar plates)	Increase in *Bifidobacterium lactis*	(94)
Unidentified floral source (India)	Viable colony counts on agar plates using bifidobacteria isolated from infant fecal samples, and identified *via* phenotypic and molecular (PCR) methods	Increase in all *Bifidobacterium* isolates	(124)
Sourwood, alfalfa, and sage (Unspecified)	Microbroth dilution, with growth measured as optical density (turbidity)	Increase in five *Bifidobacterium* species of human intestinal origin (*B. longum, B. adolescentis, B. breve, B. bifidum*, and *B. infantis*) Inhibition of *C. perfringens* and *E. aerofaciens.*	(96)
Unidentified floral source (Jordan)	Colony counts (CFU/ml) calculated from optical density (turbidity) readings	Significant increase in *Bifidobacterium infantis* and *Lactobacillus acidophilus* of intestinal origin	(97)
Tualang and multifloral (Malaysia)	Honey samples pre-treated to remove simple sugars, remaining fraction used to supplement skim milk; bacterial enumeration (colony counts on agar plates)	Increase in *Bifidobacterium longum* by all honey fractions with simple sugars removed	(112)
Clover (Unspecified)	Growth of probiotic pure cultures in skim milk supplemented with various sweeteners measured *via* cultural enumeration (colony counts on agar)	Honey best supports growth of probiotic strains, with significant increase in *Bifidobacterium bifidum* and *Lactobacillus acidophilus* numbers	(95)
***in vivo* and human studies**			
Generic, unknown floral source (India)	Wistar strain male albino rats (*n* = 36); small and large intestine collection, suspension and viable cell count	Increase in *Lactobacillus acidophilus* and *Lactobacillus plantarum*	(87)
Cotton (Egypt)	Swiss male albino mice (*n* = 42); cecum content collection, viable cell counts (bacterial enumeration on agar) of colonic bacteria	Increase in *Bifidobacterium* and *Lactobacilli*	(89)
Jarrah (Australian floral source, purchased in China)	BALB/c mice (n = 30); 16S rRNA sequencing of V3–V4 region Fecal water content measured *via* weighing fecal samples before and after drying	Gut microbiota equilibrium re-established, specifically by increasing abundance of key bacterial groups in the gut, and suppressing harmful bacteria Improvement in fecal water content, linked to alleviation of constipation	(90)
*Prunella vulgaris*, common name ‘self-heal’ (China)	Sprague Dawley male rats (*n* = 24) with induced colitis; histological analysis of colon samples, intestinal mRNA analysis, gut microbial community analysis (from caeca) *via* 16S rRNA sequencing of the V3–V4 region	Decrease in Bacteroidetes, and increase in Firmicutes; and at genus level increases in the beneficial *Lactobacillus* spp., and decrease in *Lachnospiraceae*, which is associated with the pathological features of colitis Overall reduction of symptoms associated with ulcerative colitis, mostly attributed to the abitlity of honey to modulate effects on gut microbiota	(113)
Unidentified floral source (Indonesia)	Pacific white shrimp fed honey (prebiotic), probiotic culture or synbiotic (combination of probiotic culture and honey); intestinal microbiota diversity analysis *via* DNA sequencing	Honey treatment most effective, showing increased intestinal microbiota diversity, and higher genus level abundance of beneficial (probiotic) bacteria Honey-fed shrimp showed highest survival rate post infection with *Vibrio parahaemolyticus*	(115)
Manuka (New Zealand) and multifloral (unspecified)	Pilot human clinical study where participants consumed daily dose (20 g) of honey; DNA from fecal sample sequenced for microbiota analysis	No significant changes (positive or negative) in gut microbiota populations, no antimicrobial effects of manuka honey on the beneficial populations of the gut	(119)

There is significant evidence of the prebiotic potential of honey from *in vitro* studies that assess the effect of honey on the growth of probiotic bacteria (100–107) and in probiotic food products, such as milk or yogurt, supplemented with honey (108–111). Numerous studies show that honey supports and promotes the growth of probiotic *Bifidobacterium* and *Lactobacillus* species, including *B. longum*, *B. adolescentis*, *B. breve*, *B. bifidum*, and *B. infantis, Lactobacillus. acidophilus, Lactobacillus plantarum, Lactobacillus reuteri*, and *Lactobacillus rhamnosus* (103–107, 113). The growth-promoting effect of honey on bifidobacteria and lactobacilli is usually comparable to that of oligosaccharide prebiotics, including fructooligosaccharide (FOS), galactooligosaccharide (GOS), or inulin, where these prebiotics are included as controls (42, 104, 105, 110, 112, 113). Other studies have shown that honey not only promotes the growth of probiotic cultures but has a positive effect on the metabolism of bacterial strains from the human gut (95).

As oligosaccharide composition can affect prebiotic activity, it is not surprising that different honeys can have different prebiotic properties (114). Honey can contain source-specific oligosaccharides (99)for example, native New Zealand honeys showed high levels of isomaltose and melezitose (114, 115), while raffinose was reported in Italian honey (116); and also different concentrations of commonly occurring oligosaccharides (107) influencing their prebiotic potential.

Oligosaccharides isolated from honeydew had a positive impact on the growth of fecal bacteria, specifically by promoting the populations of the beneficial bifidobacteria and lactobacilli, and by reducing the numbers of the potentially harmful *Bacteroides* and clostridia (100), quantified by the prebiotic index that scores the ratio of potentially beneficial vs. harmful bacteria relative to the overall changes (117). The prebiotic index for the honey-derived oligosaccharides was similar to that of the commercial prebiotic, FOS. Similarly, three Malaysian Tualang honeys that had been pre-treated to remove simple sugars supported enhanced growth of the probiotic *Bifidobacterium longum* (118).

### Evidence From Animal Studies and Pilot Human Trials

Numerous *in vivo* studies using animal models show that honey acts as a prebiotic, specifically by promoting the populations of probiotic bacteria, including *Bifidobacterium* spp. and *Lactobacillus* spp., (104, 106, 107, 119), and alleviating symptoms of constipation and ulcerative colitis (107, 119). The prebiotic effect of honey has also been reported in shrimp, where honey promoted the growth of known probiotics *Microbavterium* spp., *Lactobacillus* spp., and *Neptumonas* spp. (120). Shrimp receiving the honey prebiotic also had a higher abundance of gut microbes than the control or shrimp receiving either a probiotic or synbiotic. Another study investigating the prebiotic effect of honey on pacific white shrimp with *Vibrio parahaemolyticus* infection showed that those that were fed honey during the infection phase had a reduced pathogen load and higher survival rate compared to the control (no treatment) group (121).

The anti-inflammatory effect of honey can also contribute to its overall prebiotic potential, as many conditions in the gut (regardless of infection state) involve inflammation of the bowels. Various studies on the anti-inflammatory properties of honey, spanning both the gut and wound environment, suggest that honey promotes the upregulation of anti-inflammatory cytokines and downregulation of pro-inflammatory cytokines (38, 52, 53, 122, 123). In rats with acetic acid-induced gastric ulcers, a significant increase in the presence of pro-inflammatory cytokines tumor necrosis factor (TNF)-α, interleukin (IL)1-β, and IL-6 was noted. Following administration of manuka honey treatment, cytokine levels significantly decreased, the ulcers healed faster, and oxidative damage caused by acetic acid was reversed compared to the control group (122). Similarly, rats with dextran sodium sulfate-induced ulcerative colitis had a significant reduction in IL-1β and IL-6 in serum and TNF-α in colonic tissue samples after administration of Egyptian honey (124). The mechanisms suggested for inflammation reduction by honey include inhibition of reactive oxygen species, inhibition of leukocyte infiltration, inhibition of cyclooxygenase-1 and 2 (COX-1 and COX-2), and inducible nitric oxide synthase expression (53, 123). The main components in honey responsible for the anti-inflammatory and related antioxidant effects are the polyphenols, and polyphenols found in honey have been shown to alter the gut microbiome in rats with ulcerative colitis, showing both a reduction in inflammation and suppression of the populations of the potentially harmful organisms (54).

To date, there has been one human clinical study investigating the effect of daily honey consumption – specifically looking at the safety of eating manuka honey with high antibacterial activity compared to multi-floral honey (125). No significant changes in the numbers of five major bacterial groups in the gut were found, however, measuring prebiotic activity was not a primary aim of the study and the authors noted that any effects may have been masked due to interactions with other dietary components, the dose of honey used, as well as honey and storage conditions.

## Gaps and Emerging Opportunities in the Study of Prebiotic Honey

Despite current marketing and increased consumer interest around “prebiotic honey,” there are limited published studies and human response data in this research area. The bioactive components in honey responsible for its prebiotic effect have not been fully identified. Additionally, whether honey can act as a prebiotic to remediate the gut microbiome in a state of dysbiosis, such as during infection or when the bowels are inflamed, is not well understood.

Although the variable composition and therapeutic properties of honey complicate mechanistic studies of its bioactivity, it provides the opportunity for a targeted approach for different health purposes, particularly given the antimicrobial, anti-inflammatory, and prebiotic potential of honey. These bioactivities can be aligned with the emerging area of personalized medicine, which focuses on enabling more targeted therapeutic treatment and preventative options for individuals (126).

Many chronic gut-related conditions, such as irritable bowel syndrome, colon cancer, Crohn’s disease, and *C. difficile* infection, are known to be exacerbated by inflammation of the bowels (127–129). Current therapies, in particular for irritable bowel syndrome and inflammatory bowel disease, include reducing foods that contribute to inflammation. The antibacterial and anti-inflammatory activity of honey is well documented throughout the literature (19, 33) and this combined with a prebiotic activity could place honey as a suitable treatment option to benefit the microbiota and reduce inflammation of the gut. As the health of gut microbiota is a key element in understanding whole-body health and is readily manipulated, targeted dietary interventions that alter the microbiome represent a strategy of significant benefit. Honey represents an attractive option in this space and with further validation could provide a means to benefit the gut microbiome in a healthy state and to remediate the microbiome from a dysbiotic state.

## Author Contributions

All authors listed have made a substantial, direct, and intellectual contributions to the work, and approved it for publication.

## Conflict of Interest

The authors declare that the research was conducted in the absence of any commercial or financial relationships that could be construed as a potential conflict of interest.

## Publisher’s Note

All claims expressed in this article are solely those of the authors and do not necessarily represent those of their affiliated organizations, or those of the publisher, the editors and the reviewers. Any product that may be evaluated in this article, or claim that may be made by its manufacturer, is not guaranteed or endorsed by the publisher.
